# Effect of *ABCB1* genetic polymorphisms on the transport of rivaroxaban in HEK293 recombinant cell lines

**DOI:** 10.1038/s41598-018-28622-4

**Published:** 2018-07-12

**Authors:** Anne-Laure Sennesael, Nadtha Panin, Christelle Vancraeynest, Lionel Pochet, Anne Spinewine, Vincent Haufroid, Laure Elens

**Affiliations:** 1 Clinical Pharmacy Research Group, Louvain Drug Research Institute, Université catholique de Louvain (UCL), Brussels, Belgium; 2 Department of Pharmacy, Namur Research Institute for LIfe Sciences, Namur Thrombosis and Hemostasis Center (NTHC), University of Namur, Namur, Belgium; 3 Louvain Centre for Toxicology and Applied Pharmacology, Institut de Recherche Expérimentale et Clinique, UCL, Brussels, Belgium; 4 Department of Pharmacy, NTHC, CHU UCL Namur, Yvoir, Belgium; 5 Department of Clinical Chemistry, Cliniques Universitaires Saint-Luc, UCL, Brussels, Belgium; 6 Integrated PharmacoMetrics, PharmacoGenomics and PharmacoKinetics, Louvain Drug Research Institute, UCL, Brussels, Belgium

## Abstract

Direct oral anticoagulants (DOAC) are substrates for the ABCB1 transporter (also called P-glycoprotein), an active efflux pump. *ABCB1* polymorphisms have been previously reported to influence the pharmacokinetics of several drugs such as immunosuppressants and tyrosine kinase inhibitors. Recently, *in vivo* studies have suggested that genetic variants might contribute to the inter-individual variability in DOAC plasma concentrations. Therefore, we evaluated the *in vitro* effect of the most common coding *ABCB1* single nucleotide polymorphisms (SNP), 1236 C > T-2677G > T-3435C > T, and the coding *ABCB1* 1199 G > A SNP on the transport activity towards rivaroxaban. HEK293 cells were transfected to overexpress the ABCB1 wild-type (1236C-2677G-3435C, 1199 G) or variant proteins (1236C-2677G-3435T, 1236T-2677T-3435T or 1199 A). ABCB1 expression decreased the intracellular accumulation of rivaroxaban, when compared to control cells. This confirms the involvement of ABCB1 in the active transport of rivaroxaban. However, the *ABCB1* 1236 C > T-2677G > T-3435C > T and 1199 G > A SNPs had no significant influence on the intracellular accumulation of rivaroxaban when compared to the wild-type protein. These results suggest that the *ABCB1* coding SNPs investigated in the present study are unlikely to contribute to the inter-individual variability in rivaroxaban plasma concentrations.

## Introduction

The landscape of oral anticoagulant therapy has significantly changed in the last years. Direct oral anticoagulants (DOAC) have been approved for stroke prevention in non-valvular atrial fibrillation and the treatment as well as the secondary prevention of venous thromboembolism. They are increasingly used in clinical practice, partly due to their higher convenience for clinicians and patients (fixed-dose regimen, fewer interactions with drugs and food) compared with vitamin K antagonists (VKA)^1^. Four DOACs are currently available: three direct factor Xa inhibitors (apixaban, edoxaban, rivaroxaban) and one direct thrombin inhibitor (dabigatran etexilate). In 2016, guidelines issued by the European Society of Cardiology or the American College of Chest Physicians have recommended DOACs over VKAs in eligible patients^2,3^.

All DOACs are substrates for the ABCB1 transporter, also called P-glycoprotein (P-gp) or formerly designated as the multidrug resistance 1 (MDR1) protein^4^. This active efflux pump belongs to the ATP-binding cassette transporter superfamily and is involved in the disposition of multiple drugs from diverse classes such as anticancer agents, immunosuppressants or antibiotics^5^. ABCB1 expression at the apical membrane of enterocytes limits absorption, while its localization on the luminal membrane of hepatocytes and renal tubular cells enhances biliary and renal excretion respectively. In patients taking rivaroxaban, the ABCB1 protein expression in renal tubular cells is particularly important given that more than one third of the dose is eliminated unchanged in the urine^6^. Many medications that are commonly prescribed in patients with atrial fibrillation (AF) can inhibit or induce ABCB1 activity and thereby influence DOAC pharmacokinetics^7^. For instance, it has been demonstrated that the concurrent use of moderate inhibitors of ABCB1 such as amiodarone or verapamil led to a nearly 40% increase in rivaroxaban exposure^8,9^. Combining this with renal impairment or older age, two common characteristics in AF patients, produced even stronger effect on rivaroxaban pharmacokinetics.

Genetic polymorphisms have also been reported to influence ABCB1 activity and/or expression^5^. The *ABCB1* gene, located on chromosome 7, is composed of 29 exons in a 251.3-kb genomic region. More than 60 coding single nucleotide polymorphisms (SNP) have been described for *ABCB1*^10^. The three most common SNPs in the coding region are rs1128503 (1236 C > T, Gly412Gly), rs2032582 (2677 G > T, Ala893Ser) and rs1045642 (3435 C > T, Ile1145Ile). They are in strong linkage disequilibrium and present a minor allelic frequency around 50% in the Caucasian population. They have been widely investigated, with inconsistent effects on drug disposition, drug response and toxicity^5^. Another SNP of interest is the 1199 G > A non-synonymous coding SNP (rs2229109), with an allelic frequency around 6% in Caucasians. The amino acid change (Ser400Asn) caused by this SNP is located in a cytoplasmic loop involved in substrate recognition. In previous *in vitro* studies, it has been shown that 1199 G > A affects the transport of tacrolimus, vinblastine or tyrosine kinase inhibitors but that cyclosporine A, Rh123 and doxorubicin are transported in a similar extent by the wild-type and variant ABCB1 proteins^11,12^.

Despite the fixed-dose regimen of DOACs, significant inter-individual variability in peak and trough plasma concentrations has been described^13,14^. In a prospective cohort study, 40% of patients with atrial fibrillation had rivaroxaban plasma measurements outside the 5^th^–95^th^ percentile interval observed in phase 3 trials^15^. Moreover, in an analysis of the ROCKET-AF trial, rivaroxaban exposure predicted the risk of major bleeding^16^. Inversely, it was recently highlighted that DOAC patients experiencing thromboembolic events had lower plasma levels in the first month of treatment^17^. Genetic polymorphisms have been proposed to explain why patients taking the same DOAC dose present highly variable plasma concentrations^18^. Therefore, this study aimed to evaluate the in *vitro* effect of the *ABCB1* 1236 C > T-2677G > T-3435C > T and 1199 G > A SNPs on the transport activity towards rivaroxaban.

## Results

### Generation of ABCB1 recombinant cell lines

**Figure 1:**
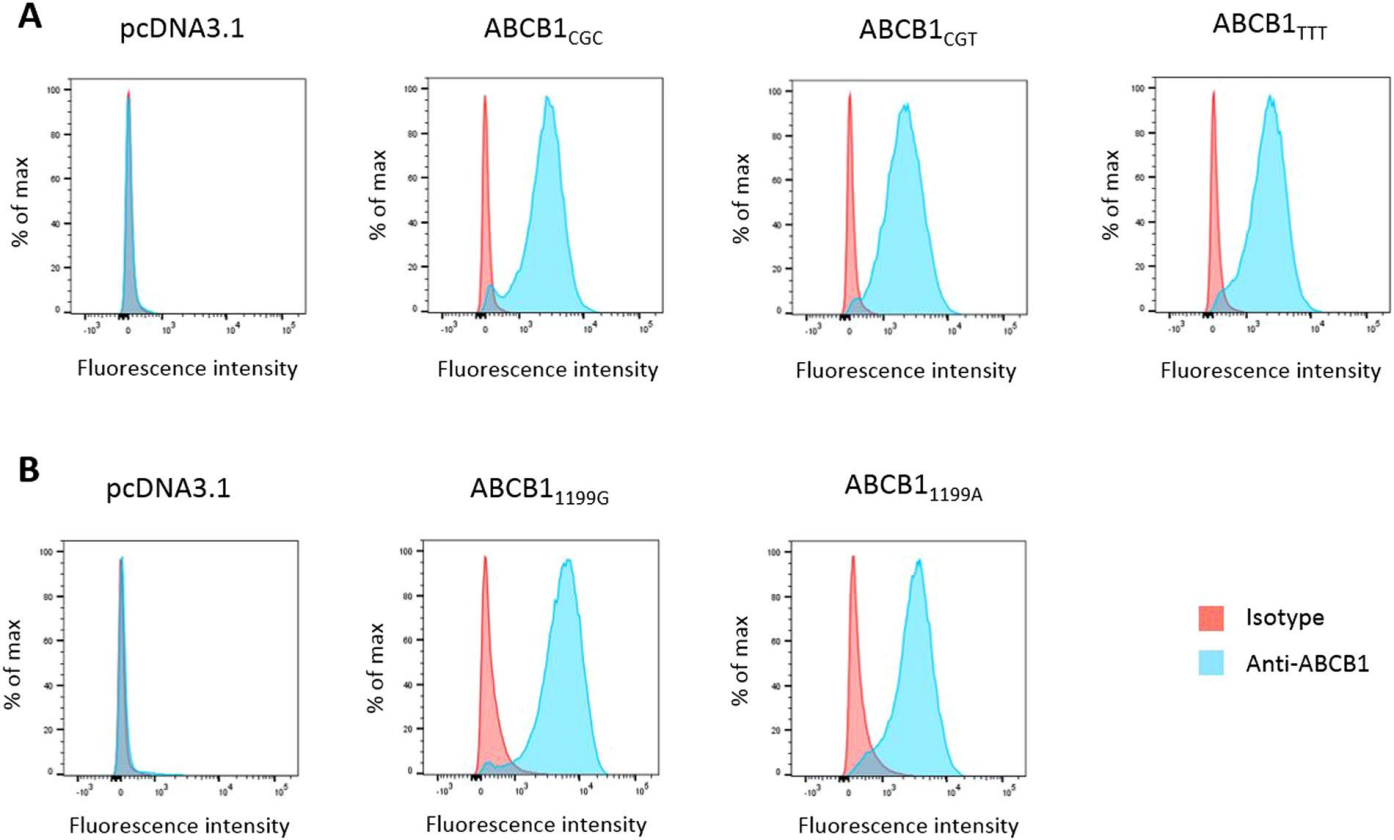
ABCB1 cell surface expression. Flow cytometry histograms of HEK293 cells transfected with (**A**) the empty pcDNA3.1 vector, *ABCB1*_CGC_, *ABCB1*_CGT_ or *ABCB1*_TTT_ and (**B**) the empty pcDNA3.1 vector, *ABCB1*_1199G_, *ABCB1*_1199A_. Cells were incubated with a FITC anti-ABCB1 antibody (blue) or a matched isotypic control (red).

HEK293 cells overexpressing ABCB1 wild-type and variant proteins have been previously generated and characterized^11,19^. For *ABCB1* 1236 C > T-2677G > T-3435C > T, the recombinant models used consisted of stably transfected cell lines with the pcDNA3.1 empty vector, HEK_pcDNA3.1_, the wild-type vector, HEK_1236C-2677G-3435C_ or two different combinations of the variant cDNA, HEK_1236C-2677G-3435T_ and HEK_1236T-2677T-3435T_, hereafter referred to as HEK_control_, HEK_CGC_, HEK_CGT_ and HEK_TTT_ respectively. For *ABCB1* 1199 G > A, three recombinant cell lines were used: HEK_control_, HEK_1199G_ (wild-type protein) and HEK_1199A_ (variant protein). To ensure similar ABCB1 surface expression among the different cell lines, cells were sorted using fluorescence activated cell sorting (FACS) with fluorescence parameters gated on the same level of intensity. As shown in Fig. 1A, similar ABCB1 surface expression was observed by analytical flow cytometry among recombinant cell lines, both for the *ABCB1* 1236 C > T-2677G > T-3435C > T and 1199 G > A SNPs. In contrast, there was no fluorescence detected in HEK_control_ ensuring no or at least very low basal expression in our negative controls.

### Impact of *ABCB1* 1236 C > T-2677G > T-3435C > T polymorphisms on the intracellular accumulation of rivaroxaban

**Figure 2:**
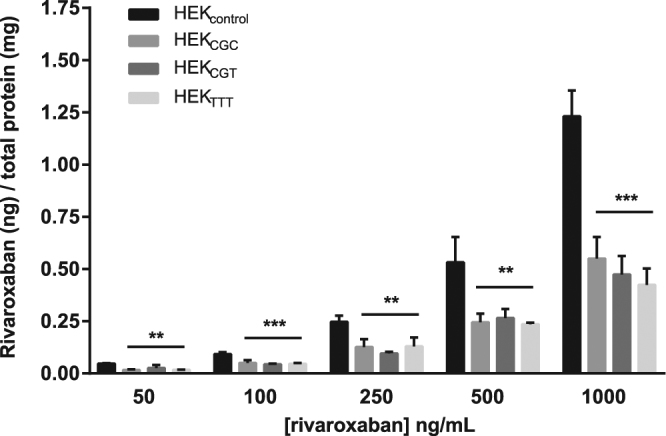
Impact of *ABCB1* 1236 C > T-2677G > T-3435C > T on the intracellular accumulation of rivaroxaban. Intracellular accumulation of rivaroxaban after 120 min of incubation (N = 3) at different concentrations in HEK_control_, HEK_CGC_, HEK_CGT_ and HEK_TTT_. The absolute amount of rivaroxaban (in ng) was divided by the total amount of proteins in cell extracts (in mg). *Compared to control cells: *p < 0.05, **p < 0.01, ***p < 0.001.

First, we assessed the impact of *ABCB1* 1236 C > T-2677G > T-3435C > T on the ABCB1 transport activity towards rivaroxaban. HEK_control_, HEK_CGC_, HEK_CGT_ and HEK_TTT_ were incubated with different concentrations of rivaroxaban ranging from 50 to 1000 ng/ml. These concentrations were chosen to cover rivaroxaban plasma levels encountered in clinical practice (concentrations up to 600 ng/ml were described in real-life patients with AF)^14,15^. As shown in Fig. 2, the intracellular accumulation of rivaroxaban was significantly decreased in recombinant cell lines overexpressing ABCB1 when compared to control cells (Fig. 2, [50, 250 and 500 ng/ml], p < 0.01; [100 and 1000 ng/ml], p < 0.001). However, we observed that the intracellular accumulation of rivaroxaban was similar between HEK_CGC_, HEK_CGT_ and HEK_TTT_ (Fig. 2, p > 0.05 at all concentrations).

### Impact of *ABCB1* 1199 G > A polymorphism on the intracellular accumulation of rivaroxaban

**Figure 3:**
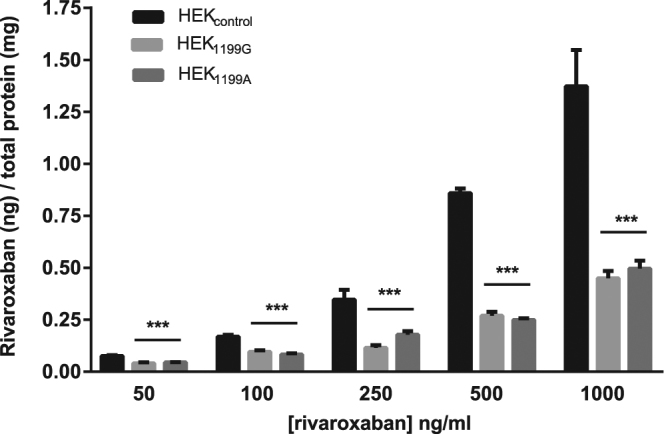
Impact of *ABCB1* 1199 G > A on the intracellular accumulation of rivaroxaban. Intracellular accumulation of rivaroxaban after 120 min of incubation (N = 3) at different concentrations in HEK_control_, HEK_1199G_ and HEK_1199A_. The absolute amount of rivaroxaban (in ng) was divided by the total amount of proteins in cell extracts (in mg). *Compared to control cells: *p < 0.05, **p < 0.01, ***p < 0.001.

Same experiments were conducted to investigate the impact of *ABCB1* 1199 G > A on the ABCB1 activity towards rivaroxaban. Again, the intracellular accumulation of rivaroxaban was lower in cell lines overexpressing ABCB1, when compared with control cells whatever their genotype (Fig. 3, [from 50 to 1000 ng/ml], p < 0.001). As for the three other variants, we did not find any statistical difference in the intracellular accumulation of rivaroxaban between cells overexpressing the ABCB1 1199 G and 1199 A proteins (Fig. 3, p > 0.05 at all concentrations).

## Discussion

In this study, we investigated the *in vitro* impact of *ABCB1* genetic polymorphisms on the transport activity towards rivaroxaban. Previously validated HEK293 recombinant cell models were used^11,19^. We confirmed the involvement of ABCB1 in the active transport of rivaroxaban^6^. Indeed, we found that ABCB1 expression decreases the intracellular accumulation of the drug. However, we failed to show a significant effect of the different SNPs investigated. The *ABCB1* 1236 C > T-2677G > T-3435C > T and 1199 G > A SNPs had no significant influence on the intracellular accumulation of rivaroxaban when compared to the wild-type protein.

To our knowledge, this is the first *in vitro* study to explore the role of *ABCB1* genetic determinants in the disposition of DOACs. Few *in vivo* studies have been published so far. Recently, a case has been described reporting a rivaroxaban-treated patient admitted at the hospital for severe anemia related to gastrointestinal bleeding^20^. This patient presented abnormally high levels of anti-Xa activity and rivaroxaban plasma concentrations as well as an unexpected delayed clearance. The authors suggested that the homozygous presence of *ABCB1* variant alleles 2677–3435TT might have contributed to the altered drug clearance. This observation seems in contradiction with our results showing no influence of those SNPs using *in vitro* models. However, it must be stressed that this observation relates to an isolated clinical case and, given the relatively high frequency of the homozygous genotype (up to 25% of the population of Caucasian origin is expected to be 2677–3435TT), it might simply reflect a spurious association that is only due to chance. Furthermore, this patient received concomitant simvastatin (40 mg q.d) which is known to inhibit CYP3A4 enzyme but also the ABCB1 and ABCG2 transporters and might have explained the findings. Finally, the patient presented a moderate renal impairment that could have worsened the renal elimination of the drug.

More recently, a randomized, two-center, crossover study was performed in healthy volunteers to study the influence of *ABCB1* polymorphisms on the pharmacokinetics of dabigatran and rivaroxaban. Peak plasma concentration and area under the curve (AUC) were compared across three different *ABCB1* genotypes i.e. 2677GG-3435CC, 2677GT-3435CT and 2677TT-3435TT and the effect of clarithromycin co-administration on the pharmacokinetics was also assessed. In agreement with the fact that rivaroxaban and dabigatran etexilate are both substrates for ABCB1, clarithromycin co-administration led to a two-fold increase in both drugs’ AUC, irrespective of *ABCB1* genotype. Their conclusion meets the observations made in the present investigation as they inferred that *ABCB1* genotype is not a significant determinant of inter-individual variability in dabigatran and rivaroxaban pharmacokinetics but that co-administration of a P-gp/CYP3A4 inhibitor with dabigatran etexilate or rivaroxaban may warrant caution.

ABCB1 is involved in the transport of many endogenous, dietary and drug compounds that do not share obvious common structural features. The *ABCB1* 1236 C > T-2677G > T-3435C > T SNPs have been inconstantly described to influence the clinical pharmacokinetics of immunosuppressants, anticancer agents, antiepileptic drugs or antidepressants^5^. A trend towards higher concentrations in patients carrying the variant haplotype seems to emerge, but therapy adjustments are currently not required or even recommended. The *ABCB1* 1199 G > A SNP has previously shown a substrate-dependent impact on transport activity. For instance, the variant allele (1199 G) decreased ABCB1 activity towards tacrolimus in HEK293 and K562 recombinant cell lines, but did not affect the transport of cyclosporine^11^. Similarly, the *in vitro* effect of 1199 G > A was lower for nilotinib compared to imatinib, two tyrosine kinase inhibitors^12^. Structural flexibility and multiple binding sites were suggested as explanations for the polyspecificity of ABCB1^21^. In that way the 1199 G > A SNP, which is located in a cytoplasmic loop involved in substrate recognition, may affect the binding of some ABCB1 substrates while the transport of the others remains unchanged as this seems to be the case for rivaroxaban^11^.

Another explanation for our negative findings may rely in the fact that rivaroxaban is described as a weak to moderate substrate for ABCB1^4,22^. In Caco-2 cells for instance, ABCB1 inhibition reduced rivaroxaban efflux by only 23% whereas the efflux of dabigatran was inhibited by 87%^4^. Moreover, rivaroxaban is also substrate for the ABCG2 transporter, known as breast cancer resistance protein (BCRP), and the cytochrome P450 (CYP) 3A4/3A5 enzyme. The active renal secretion by ABCB1 and ABCG2 and hepatic transformation by CYP3A4/3A5 account for 30% and 18% of total drug elimination respectively^6^. Although combined ABCB1, ABCG2 and CYP3A4 inhibitors were shown to increase rivaroxaban plasma concentrations by up to 150%, the impact of ABCB1 inhibition alone seems marginal^23,24^. Gong and colleagues demonstrated that rivaroxaban clearance was significantly reduced in mice lacking both ABCB1 and ABCG2, but did not differ for individual transporter knockout^25^. This suggests a compensatory role of one transporter when the other is inhibited. It is important to note that, in HEK293 cells, the endogenous expression of ABCB1 and ABCG2 is reported to be very low^26^.

Other genetic determinants could contribute to the inter-individual variability in DOAC plasma concentrations. Future studies are needed to assess the impact of *ABCG2* polymorphisms on the transport of rivaroxaban. The *ABCG2* rs2231142 (421 C > A, Gln141Lys) SNP, with an allelic frequency around 5–10% in Caucasians and 30–60% in East Asians, is of particular interest as it significantly affected plasma trough concentrations in patients treated with apixaban^27,28^. In the same study the CYP3A5*3 allele, which is highly prevalent in Caucasians, also contributed to higher apixaban levels. Finally, it could be relevant to investigate the CYP3A4*22 allele, which was associated with a lower CYP3A4 expression and/or activity^29^.

In summary, the *ABCB1* 1236 C > T-2677G > T-3435C > T and 1199 G > A SNPs did not affect the efflux of rivaroxaban in HEK293 recombinant cell lines. Therefore, we believe that these SNPs are unlikely to contribute to the inter-individual variability in rivaroxaban plasma concentrations. Future studies should focus on the role of *ABCG2*, *CYP3A4* and *CYP3A5* genetic polymorphisms in the transport of rivaroxaban.

## Materials and Methods

### Materials

Rivaroxaban and [^13^C6]-rivaroxaban-d5 were purchased from Alsachim (Strasbourg, France).

G418 was purchased from Roche Applied Science (Vilvoorde, Belgium).

### Cell culture

Human Embryonic Kidney (HEK293) cells were grown in Dulbecco’s Modified Eagle medium (DMEM) Glutamax 4.5 g/l glucose (Gibco, Invitrogen) supplemented with 10% (v/v) of Fetal Bovin Serum (Gibco, Invitrogen) and 1% (v/v) of Penicillin-Streptomycin solution (Gibco, Invitrogen) at a temperature of 37 °C in the presence of 5% of CO_2_.

### Generation of stable recombinant cell lines

The generation of stable recombinant cell lines has been described in previous studies^11,19^. Briefly, the expression vectors pcDNA3.1 with cDNA encoding ABCB1 (*ABCB1*_1199G_ and *ABCB1*_1236T-2677T-3435T_) were used. Mutated plasmids *ABCB1*_1199A_, *ABCB1*_1236C-2677G-3435C_ and *ABCB1*_1236C-2677G-3435T_ were obtained by site-directed mutagenesis. Plasmids were sequenced to confirm the presence of mutations and HEK293 cells were then transfected with pcDNA3.1 vectors. G418 (concentration 1 mg/ml) was added to select stable recombinant cell lines, which were characterized by flow cytometry, western-blot and immunofluorescence.

### Characterization of ABCB1 cell surface expression

After one week of culture in the presence of G418, ABCB1 cell surface protein expression was characterized by flow cytometry as previously described with minor changes^11^. For each recombinant cell line, 10^6^ cells were harvested by centrifugation and washed twice with ice-cold buffer solution (phosphate buffer saline (PBS), FBS 1%, EDTA 1 mM). Cells were then incubated 45 min on ice in the dark in buffer solution containing FITC Mouse anti-P-glycoprotein antibody diluted 1:10 (clone17F9 557002, BD Pharmingen) or its isotypic control diluted 1:10 (FITC Mouse IgG 2bk, clone27-35 555742, BD Pharmingen). Cells were finally washed with ice-cold buffer solution, centrifuged and resuspended in buffer solution. Samples were analyzed using Fluorescence-activated cell sorting (FACS) Canto II (BD).

### Rivaroxaban accumulation

Rivaroxaban accumulation experiments were performed as previously described for tacrolimus or cyclosporin A^11^. One day before the experiment, 350.000 cells were seeded in poly-L-lysine-coated 24-well plates in 500 µl of complete medium. Rivaroxaban was added at 5 different concentrations: 50, 100, 250, 500 and 1000 ng/ml. Recombinant cells were incubated for 120 min at 37 °C, 5% of CO_2_^22^. Cells were then washed twice with ice-cold PBS. Cold conditions were applied to block enzymatic activity and to avoid active transport out of the cells, thereby preventing any drug loss during washing steps. After centrifugation and removal of the supernatant, cells were detached with ice-cold PBS containing 0.2% EDTA. Cell pellets were conserved at −80 °C until quantification by LC-MS/MS analysis.

### Rivaroxaban quantification

The amount of rivaroxaban extracted from the cell pellet was quantified by LC-MS/MS analysis according to a previously described method, with minor adaptive changes in the sample preparation^30^. The chromatography system was made up of an UPLC Acquity H-Class system (Waters) coupled with a tandem-quadrupole mass spectrometer Xevo TQ-S (Waters). Separation of the analytes was achieved on a Waters Cortecs UPLC C18 column (2.1 × 100 mm, 1.6 µm). Rivaroxaban was extracted with 100 µl of methanol and water (volume ratio 7:3) containing the internal standard ([^13^C6]-rivaroxaban-d5) at 2 ng/ml. Blank cell pellets were prepared similarly for the calibration curve, with extraction solutions containing rivaroxaban at concentrations ranging from 0.125 ng/ml to 50 ng/ml. Samples were vortexed and then sonicated for 10 minutes in an ultrasound bath. After centrifugation at 20,000 g for 10 minutes, the supernatant was transferred to a HPLC vial (Macheray-Nagel, Düren, Germany). An aliquot (2 µL) of the final extract was injected into the LC-MS/MS system. For each experiment, the absolute amount of rivaroxaban recovered from the cell extracts was normalized to the amount of proteins measured with the BCA kit (Thermoscientific, Ghent, Belgium).

### Statistical analysis

All experiments were repeated twice. Statistical analyses were performed using GraphPad Prism 7.0 for Windows (San Diego California, USA). Analyses of variance were used under the null hypothesis that the means of the different groups were equal. When differences among means were significant, post-hoc Student-Newman-Keuls tests were carried out. In all cases, a p-value of less than 0.05 was considered statistically significant.

### Data availability

All data generated or analyzed during the current study are available from the corresponding author on reasonable request.
